# Clinical characterization of chromosome 5q21.1–21.3 microduplication: A case report

**DOI:** 10.1515/med-2020-0199

**Published:** 2020-11-09

**Authors:** Shuang Chen, Yang Yu, Han Zhang, Leilei Li, Yuting Jiang, Ruizhi Liu, Hongguo Zhang

**Affiliations:** Center for Reproductive Medicine and Center for Prenatal Diagnosis, First Hospital, Jilin University, 1 Xinmin Street, Chaoyang District, Changchun, Jilin Province, 130021, China

**Keywords:** chromosome 5, prenatal diagnosis, microduplication, genetic counseling

## Abstract

Chromosomal microdeletions and microduplications likely represent the main genetic etiologies for children with developmental delay or intellectual disability. Through prenatal chromosomal microarray analysis, some microdeletions or microduplications can be detected before birth to avoid unnecessary abortions or birth defects. Although some microdeletions or microduplications of chromosome 5 have been reported, numerous microduplications remain undescribed. We describe herein a case of a 30-year-old woman carrying a fetus with a chromosome 5q21.1–q21.3 microduplication. Because noninvasive prenatal testing indicated a fetal chromosome 5 abnormality, the patient underwent amniocentesis at 22 weeks 4 days of gestation. Karyotyping and chromosomal microarray analysis were performed on amniotic fluid cells. Fetal behavioral and structural abnormalities were assessed by color and pulsed Doppler ultrasound. Clinical characteristics of the newborn were assessed during the follow-up. The left lateral ventricle appeared widened on ultrasound, but the infant appeared normal at birth. The 5q21.1–q21.3 microduplication in the fetus was inherited from his mother. There are seven genes in this duplication region, but their main functions are unclear. According to this case report, microduplication in this region could represent a benign mutation. Clinicians should pay attention to the breakpoints and the genes involved when counseling patients with microdeletions and microduplications.

## 1 Introduction

Chromosomal microdeletions, microduplications, and unbalanced rearrangements represent the main genetic etiological factors for children with developmental delay or intellectual disability [1]. Currently, chromosomal microarray analysis (CMA) is considered a first-tier diagnostic tool for these children [2]. Through prenatal diagnosis of CMA, some microdeletions or microduplications can be detected before birth to avoid unnecessary abortions or birth defects [3]. The clinical features of some chromosome 5 microduplications have been described previously [4,5,6,7,8]. Jamsheer et al. [8] presented a case of bilateral radial agenesis with absent thumbs, complex heart defect, short stature, and facial dysmorphism in a 9.5-year-old patient with pure distal microduplication of 5q35.2–5q35.3. Oexle et al. [9] reported a microduplication in chromosome 5p13.1–p13.2 associated with developmental delay, macrocephaly, obesity, and lymphedema. Nonetheless, numerous microduplications remain undescribed. A greater understanding of the relationship between rare fetal genomic rearrangements and clinical phenotypes would improve genetic counseling for prenatal diagnosis. Here, we describe the clinical characterization of a case of 5q21.1–q21.3 microduplication prenatally diagnosed using a single-nucleotide polymorphism (SNP) array presenting with a normal phenotype. We review the potential function of genes in the 5q21.1–q21.3 region.

## 2 Case report

This study was approved by the Ethics Committee of the First Hospital of Jilin University (No. 2020-336), and informed written consent was obtained from the patient for the publication of this case report.

**Figure 1:**
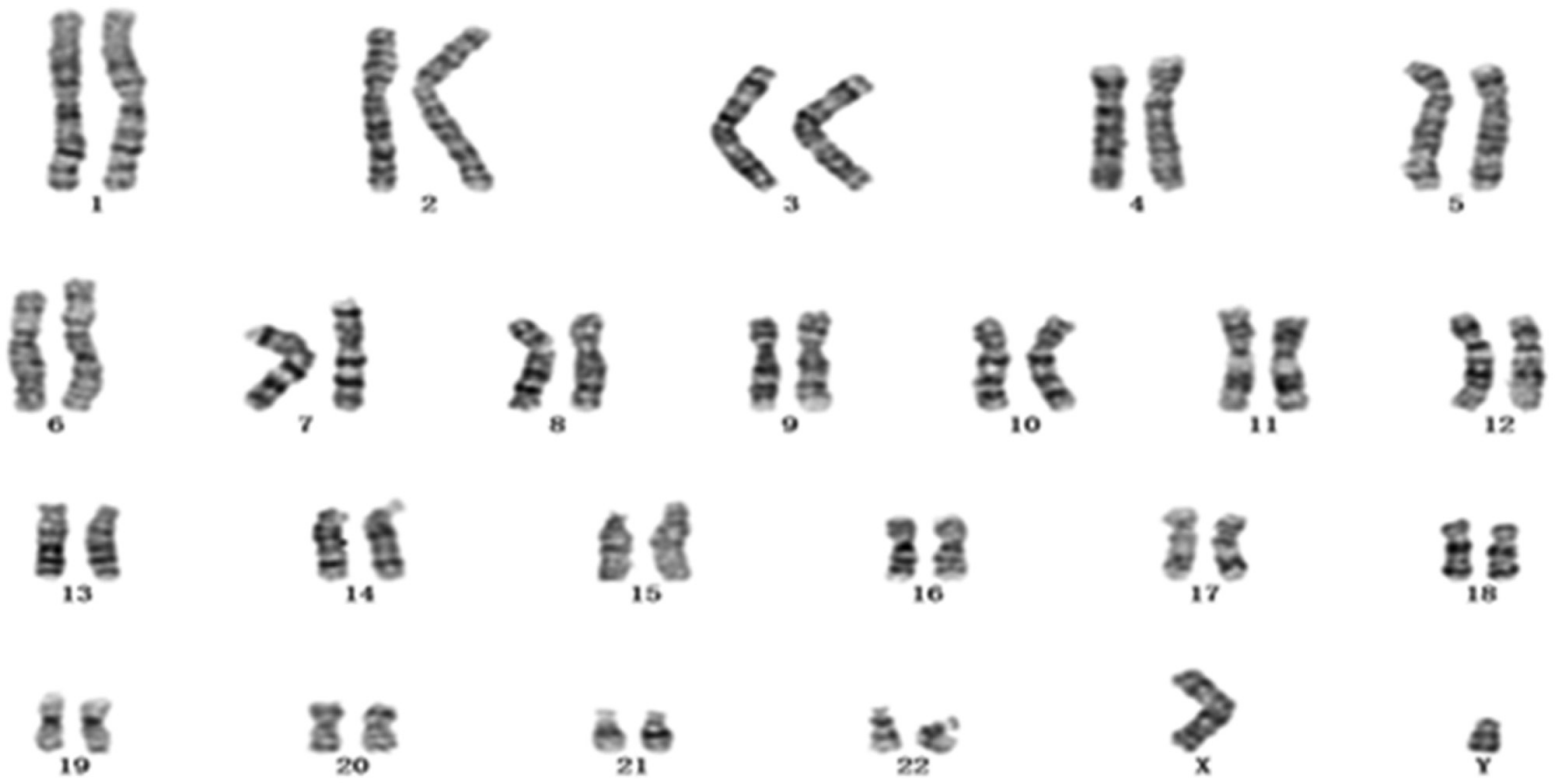
Normal karyotype (46,*XY*) of the fetus identified by GTG banding technique.

**Figure 2:**
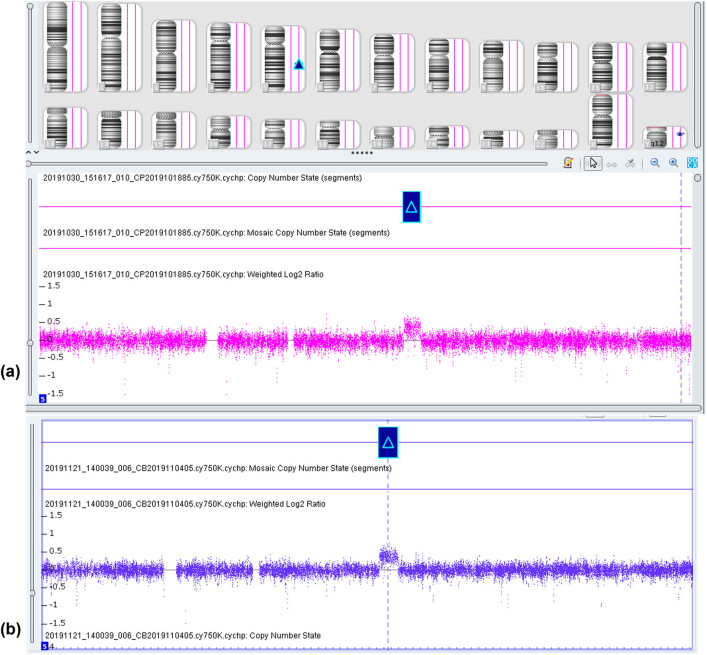
(a) Chromosomal microarray analysis (CMA) array on uncultured amniocytes depicted 5q21.1q21.3 (101116629–105838444) microduplication. (b) CMA array on peripheral blood depicted 5q21.1q21.3 (101070989–105838444).

A 30-year-old pregnant woman had previously delivered an apparently healthy girl; this was her second pregnancy. The couple was nonconsanguineous and phenotypically healthy, with no family history of diabetes mellitus or congenital malformations. The patient had a well-developed female phenotype and normal hearing. Noninvasive prenatal testing at 21 weeks 3 days of gestation indicated a fetal chromosome 5 abnormality. Amniocentesis was performed following informed consent at 22 weeks 4 days of gestation. On the day of amniocentesis, the patient underwent clinical ultrasound examination. Ultrasonographic findings indicated a normal, single, live fetus, with the following findings: biparietal diameter, 5.1 cm; head circumference, 19.5 cm; abdominal circumference, 16.7 cm; femur length, 3.4 cm; systolic-diastolic ratio, 2.64; pulsatility index, 0.94; and resistivity index, 0.62. Karyotyping and CMA were performed on amniotic fluid cells. Cytogenetic analysis showed a normal fetal karyotype, 46, *XY* (Figure 1). A 4.772 Mb microduplication of 5q21.1–q21.3(arr[GRCh37] 5q21.1q21.3(101116629–105838444) × 3) was identified by the SNP array (Figure 2a). After genetic counseling and informed consent, peripheral blood was collected from the parents for CMA analysis at 24 weeks of gestation. The CMA results of the mother showed a 4.767 Mb microduplication of 5q21.1–q21.3(arr[GRCh37] 5q21.1q21.3(101070989–105838444) × 3) (Figure 2b). The father’s CMA results were normal.

After genetic counseling, the parents chose to continue the pregnancy. Ultrasonographic findings at 30 weeks 4 days of gestation indicated a single live fetus, with a single ventricle in the intracalvarium. The skull ring was complete, the width of the right lateral ventricle was 0.4 cm, and the width of the left lateral ventricle was 0.78 cm. The biparietal diameter was 7.6 cm, head circumference was 28.2 cm, abdominal circumference was 26.5 cm, and femur length was 5.7 cm. A four-chamber tangential plane was seen, and the fetal heart rate was 148 times/min. The spine was visualized as double light bands arranged in parallel, neat, and continuous. The humerus, femur, ulna, radius, tibia, fibula, and both hands and feet were visible. The abdominal visceral section of the fetus showed stomach and bladder filling. The liver and kidneys were visible, and there was no separation of the renal pelvis. The fetal upper lip was continuous. The fetal movement was visible. Effusion could be seen in the bilateral testicular tunica vaginalis, with a width of 7 mm on the right and 6 mm on the left. The systolic–diastolic ratio was 2.8. The maximum fluid dark area of the amniotic fluid was 86 mm, and a battledore placenta was observed.

The mother chose a natural labor and delivered a male infant at 39 weeks of gestation. The baby was 50 cm long and weighed 3,100 g. A newborn hearing screening was normal. During the 6-month follow-up after birth, it was found that the child had normal phenotypes and was developing normally.

### 2.1 Cytogenetic analysis

Amniotic fluid cells were obtained through amniocentesis after obtaining written informed consent, collected by centrifugation, inoculated in flasks in accordance with laboratory standards, and cultured in CO_2_ incubators for 10 days. Chromosome analysis using GTG staining was performed similar to our previous study [4]. The karyotype was described in accordance with the International System for Human Cytogenetic Nomenclature (ISCN 2013) [10]. Twenty metaphase spreads were analyzed.

### 2.2 Chromosome microarray analysis

Genomic DNA was extracted from 10 mL of uncultured amniocytes using the QIAamp DNA Mini kit (Qiagen, Hilden, Germany) following the manufacturer’s instructions. The SNP array analysis was performed using the Human CytoScan 750K BeadChip (Affymetrix, San Diego, CA, USA). Image data were analyzed using Chromosome Analysis Suite v4.0 software (ThermoFisher Scientific, Waltham, MA, USA). The final results were analyzed using the Database of Chromosomal Imbalance and Phenotype in Humans using Ensembl Resources (DECIPHER), the Database of Genomic Variants, Online Mendelian Inheritance in Man (OMIM), and National Center for Biotechnology Information.

## 3 Discussion

Some microdeletion syndromes are associated with developmental delay or intellectual disability, but microduplications have not yet been characterized in the same way [11]. For chromosome 5, clinical features of 5q31.3 microdeletion syndrome [12], 5q11.2 microdeletion [13], 5q14.3 microdeletion [14], and 5q31.3 microdeletion syndrome [15] have been reported. However, microduplication of the long arm of chromosome 5 is extremely rare. Although microduplications of 5q35.2–q35.3 have been reported [4,6,8], chromosome 5q21.1–21.3 microduplication has not been clinically characterized. We report the prenatal diagnosis of a fetus with a 4.7 Mb microduplication of chromosome 5q21.1–q21.3, inherited from his mother.

No severely abnormal features were found in the fetus. Ultrasound examination revealed an increase in the width of the left lateral ventricle and battledore placenta; all other indicators were normal. The same microduplication was carried by the mother, and she had a normal phenotype. After genetic consultation and informed consent, the couple chose to continue the pregnancy and gave birth to a healthy infant.

To explore the relationship between gene and phenotype in the region of 5q21.2–q21.3, we searched for related genes in DECIPHER and OMIM. There are seven genes in the region of 5q21.2–q21.3(101116629–105838444). Table 1 presents these genes and the functions of their products; however, the functions and clinical phenotypes associated with these genes need further study. Yousaf et al. [16] reported a c.2510G > A transition variant in *PPIP5K2* associated with hearing loss in two large, apparently unrelated Pakistani families. Animal experiments have shown that *PPIP5K2* is expressed in the cochlear and vestibular sensory hair cells, supporting cells, and spiral ganglion neurons. Mice with a homozygous deletion of the *PPIP5K2* phosphatase domain exhibited generation of cochlear outer hair cells and elevated hearing thresholds. Therefore, the *PPIP5K2* gene has an important role in hearing in humans [16]. However, the patients carried with chromosome 5q21.1–21.3 microduplication in the current study did not have hearing problems. The effect of an additional copy of one or more related genes has not been reported. Although the patients (mother and baby) in this report had normal phenotypes, additional cases with the same microduplication need to be studied.

**Table 1:** Genes and gene product functions in the region of 5q21.2q21.3 (101116629–105838444)

Gene	OMIM	Description	Function of gene product
PPIP5K2	6,11,648	Diphosphoinositol pentakisphosphate kinase 2	Bifunctional inositol kinase that acts in concert with the IP6K kinases IP6K1, IP6K2, and IP6K3 to synthesize the diphosphate group-containing inositol pyrophosphates diphosphoinositol pentakisphosphate, PP-InsP5, and bis-diphosphoinositol tetrakisphosphate, (PP)2-InsP4.PP-InsP5 and (PP)2-InsP4regulate a variety of cellular processes, including apoptosis, vesicle trafficking, cytoskeletal dynamics, exocytosis, insulin signaling, and neutrophil activation. Required for normal hearing
SLCO4C1	6,09,013	Solute carrier organic anion transporter family, member 4C1	Organic anion transporter, capable of transporting pharmacological substances such as digoxin, ouabain, thyroxine, methotrexate, and cAMP. Involved in the uptake of the dipeptidyl peptidase-4 inhibitor sitagliptin and hence may play a role in its transport into and out of renal proximal tubule cells
SLCO6A1	6,13,365	Solute carrier organic anion transporter family member 6A1	NA
PAM	1,70,270	Peptidylglycine alpha-amidating monooxygenase	Bifunctional enzyme that catalyzes the posttranslational modification of inactive peptidylglycine precursors to the corresponding bioactive alpha-amidated peptides, a terminal modification in biosynthesis of many neural and endocrine peptides
GIN1	—	Gypsy retrotransposon integrase 1	NA
C5ORF30	6,16,608	Macrophage immunometabolism regulator	Probably plays a role in trafficking of proteins via its interaction with UNC119 and UNC119B cargo adapters: may help the release of UNC119 and UNC119B cargo or the recycling of UNC119 and UNC119B. May play a role in ciliary membrane localization via its interaction with UNC119B and protein transport into photoreceptor cells
NUDT12	6,09,232	Nudix hydrolase 12	Hydrolyzes NAD(P)H to NMNH and AMP (2′,5′-ADP), and diadenosine diphosphate to AMP. Has also activity toward NAD(P)(+), ADP-ribose and diadenosine triphosphate. May act to regulate the concentration of peroxisomal nicotinamide nucleotide cofactors required for oxidative metabolism in this organelle

NA, not applicable.

A limitation of this study is that the carrier of the microduplication is a phenotypically normal woman and the infant is male. It is unclear whether the infant will continue to develop normally, and follow-up is ongoing. This case provides important information for clinical genetic counseling.

## 4 Conclusion

We report here a case of a fetus with a prenatally diagnosed 5q21.1–q21.3 microduplication, which was inherited from his mother. Although a widened left lateral ventricle was noted on ultrasound at 30 weeks 4 days of gestation, the infant had a normal phenotype at birth. The 5q21.1–q21.3 microduplication identified here could be a benign mutation, but clinicians should pay attention to the breakpoints and the genes involved when counseling patients with microdeletions and microduplications.
